# Silencing of *RUNX2* enhances gemcitabine sensitivity of *p53*-deficient human pancreatic cancer AsPC-1 cells through the stimulation of TAp63-mediated cell death

**DOI:** 10.1038/cddiscovery.2015.10

**Published:** 2015-08-10

**Authors:** H Sugimoto, M Nakamura, H Yoda, K Hiraoka, K Shinohara, M Sang, K Fujiwara, O Shimozato, H Nagase, T Ozaki

**Affiliations:** 1 Laboratory of DNA Damage Signaling, Chiba Cancer Center Research Institute, Chuou-ku, Chiba, Japan; 2 Laboratory of Cancer Genetics, Chiba Cancer Center Research Institute, Chuou-ku, Chiba, Japan; 3 Innovative Therapy Research Group, Nihon University Research Institute of Medical Science, Nihon University School of Medicine, Itabashi, Tokyo, Japan

## Abstract

Runt-related transcription factor 2 (RUNX2) has been considered to be one of master regulators for osteoblast differentiation and bone formation. Recently, we have described that RUNX2 attenuates p53/TAp73-dependent cell death of human osteosarcoma U2OS cells bearing wild-type *p53* in response to adriamycin. In this study, we have asked whether *RUNX2* silencing could enhance gemcitabine (GEM) sensitivity of *p53*-deficient human pancreatic cancer AsPC-1 cells. Under our experimental conditions, GEM treatment increased the expression level of p53 family TAp63, whereas RUNX2 was reduced following GEM exposure, indicating that there exists an inverse relationship between the expression level of TAp63 and RUNX2 following GEM exposure. To assess whether TAp63 could be involved in the regulation of GEM sensitivity of AsPC-1 cells, small interfering RNA-mediated knockdown of *TAp63* was performed. As expected, silencing of *TAp63* significantly prohibited GEM-dependent cell death as compared with GEM-treated non-silencing cells. As *TAp63* was negatively regulated by RUNX2, we sought to examine whether RUNX2 knockdown could enhance the sensitivity to GEM. Expression analysis demonstrated that depletion of *RUNX2* apparently stimulates the expression of TAp63, as well as proteolytic cleavage of poly ADP ribose polymerase (PARP) after GEM exposure, and further augmented GEM-mediated induction of p53/TAp63-target genes, such as *p21*^*WAF1*^, *PUMA* and *NOXA*, relative to GEM-treated control-transfected cells, implying that RUNX2 has a critical role in the regulation of GEM resistance through the downregulation of TAp63. Notably, ablation of *TAp63* gave a decrease in number of γH2AX-positive cells in response to GEM relative to control-transfected cells following GEM exposure. Consistently, GEM-dependent phosphorylation of ataxia telangiectasia-mutated protein was remarkably impaired in *TAp63* knockdown cells. Collectively, our present findings strongly suggest that RUNX2-mediated repression of TAp63 contributes at least in part to GEM resistance of AsPC-1 cells, and thus silencing of *RUNX2* may be a novel strategy to enhance the efficacy of GEM in *p53*-deficient pancreatic cancer cells.

## Introduction

Human pancreatic cancer is a highly aggressive, as well as metastatic tumor, and represents the fourth and fifth leading causes of cancer-related death in the United States and Japan, respectively, whose incidence is increasing.^1,2^ Although the best chance of long-term survival is the complete surgical resection, most patients (over 80%) are not amenable to surgery at the time of diagnosis because of its difficulty in early detection.^3^ Unfortunately, even in patients who underwent surgery, a high rate of relapse with high local recurrence rates up to 50% is detectable, which leads to a 5-year survival rate of under 5%.^4^ Therefore, chemotherapy and/or radiotherapy is the only option.

For chemotherapy, the antimetabolite deoxycytidine nucleoside analog gemcitabine (GEM) is the current first line of standard treatment given to most patients with advanced pancreatic cancer.^5,6^ To achieve its anticancer activity, GEM requires phosphorylation within tumor cells to become an active form. The active one then accumulates within cancer cells and its incorporation into genome DNA correlates with its cytotoxicity.^7^ However, GEM treatment provides limited clinical benefits, especially in advanced and metastatic disease.^8^ Furthermore, a lot of clinical studies demonstrated that the combination of GEM with other cytotoxic drugs does not remarkably improve the poor prognosis of pancreatic cancer patients.^9–12^ Thus, a novel therapeutic option(s) based on biology of pancreatic cancer and also the understanding of the precise molecular mechanism(s) behind its resistance to GEM should be necessary to improve outcomes in patients with pancreatic cancer.

Mammalian RUNX family of transcription factors including runt-related transcription factor 1 (RUNX1), RUNX2 and RUNX3, is an evolutionarily conserved regulator of cell fate. Each of RUNX family members contains a highly conserved ‘runt’ domain, which recognizes and directly binds to the consensus sequences (TGTGGT or ACCACA), and mediates transcriptional activation or repression of their target genes.^13,14^ In spite of their structural similarities, RUNX family members have divergent physiological roles. It has been well established that RUNX1, RUNX2 and RUNX3 have a critical role in the regulation of hematopoiesis,^15^ bone formation^16,17^ and gastrointestinal/neuronal development,^18,19^ respectively. Meanwhile, all of these RUNX family members are also implicated in carcinogenesis. For example, *RUNX1* is a frequent target of chromosomal translocations in hematopoietic malignancies,^20^ and the loss or reduction of *RUNX3* expression can be detected in over 80% of gastric cancers.^21,22^ These observations strongly suggest that RUNX1, as well as RUNX3, acts as a putative tumor suppressor.

In a sharp contrast to RUNX1 and RUNX3, RUNX2 may have a pro-oncogenic potential. A growing body of evidence demonstrated that RUNX2 is aberrantly expressed in several human cancers including pancreatic,^23^ thyroid,^24^ breast,^25,26^ prostate,^27^ lung,^28^ colon,^29^ ovarian cancers^30^ and osteosarcoma.^31,32^ Consistent with these observations, it has been shown that RUNX2 has an ability to transactivate genes implicated in cancer cell migration and invasion.^33–38^ Indeed, Tandon *et al.* ^39^ revealed that knockdown of *RUNX2* in invasive breast cancer cells promotes cell death in response to glucose- and growth factor-deprivation. Similarly, Akech *et al.* ^40^ described that depletion of *RUNX2* in prostate cancer cells inhibits cell migration and invasion *in vitro* and RUNX2 expression in prostate cancer tissues is associated with metastasis. In addition, it has been found that there exists a positive correlation between *RUNX2* gene amplification and poor chemo-response in osteosarcoma patients.^32^ Unfortunately, the precise molecular mechanism(s) how RUNX2 could contribute to the development and progression of the above-mentioned cancers remains elusive.

The representative tumor-suppressor p53 protects normal cells from onocogenic transformation by prohibiting undesirable propagation of damaged cells. As expected from its structural property, p53 acts as a nuclear transcription factor, which transactivates numerous of its target genes implicated in the induction of cell cycle arrest, cellular senescence and/or cell death following DNA damage.^41^ Accumulating evidence strongly suggests that p53-mediated cellular processes are tightly linked to its transcriptional activity. Although extensive mutation searches revealed that *p53* is mutated in over 50% of human cancers. Among them, *p53* mutation has been detectable in approximately 75% of pancreatic cancer.^42^ As most of *p53* mutations are found within the genomic region encoding its DNA-binding domain, mutant forms of p53 lack sequence-specific transactivation ability and thereby act as dominant-negative inhibitors against wild-type p53.^41,43^ Unlike *p53*, *p73* and *p63*, which belong to p53 family, are rarely mutated in human cancers.^44^ Owing to the alternative C-terminal splicing and promoter usage, *p73* and *p63* encode multiple isoforms such as transactivating isoforms (TAp73 and TAp63) and N-terminally truncated isoforms lacking transactivation domain (ΔNp73 and ΔNp63).^45,46^ As expected from their structural similarity to p53, TAp73 and TAp63 have a fundamental role in the regulation of DNA damage response.^41^

Recently, we have demonstrated for the first time that RUNX2 attenuates p53 and/or TAp73-dependent cell death in *p53*-proficient osteosarcoma U2OS cells following DNA damage inducer adriamycin (ADR) exposure.^47,48^ In this study, we have focused on *p53*-deficient pancreatic cancer AsPC-1 cells and found that depletion of *RUNX2* enhances the sensitivity to GEM of AsPC-1 cells in association with a significant stimulation of TAp63-dependent cell death pathway.

## Results

### AsPC-1 cells are much more resistant to GEM than SW1990 cells

**Figure 1:**
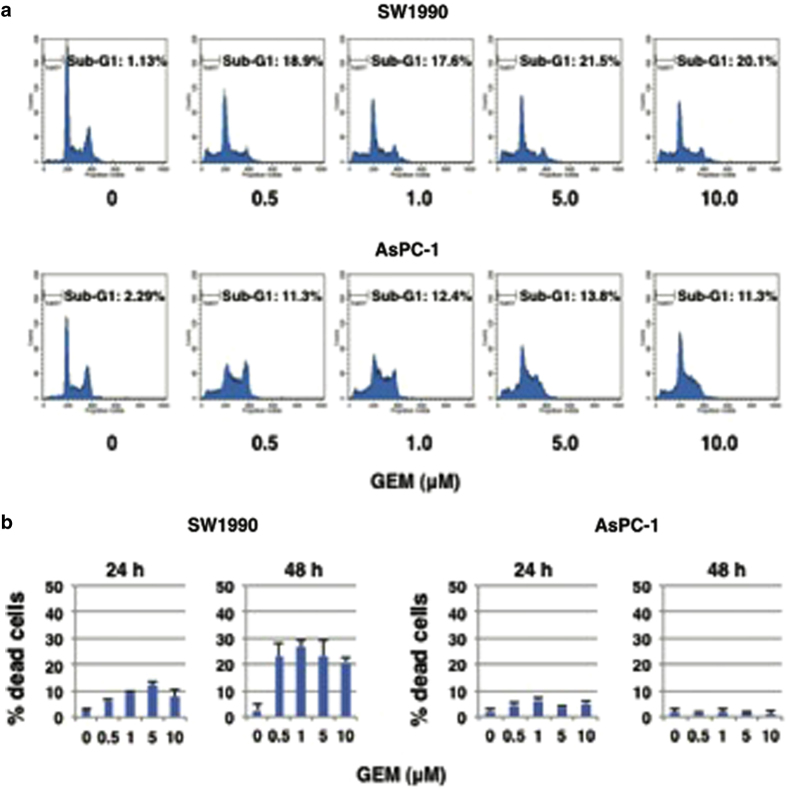
AsPC-1 cells are much more resistant to GEM than SW1990 cells. (**a**) FACS analysis. SW1990 (upper panels) and AsPC-1 (lower panels) cells were exposed to the indicated concentrations of GEM. Forty-eight hours after treatment, floating and attached cells were collected and their cell cycle distribution was analyzed by FACS. (**b**) Trypan blue exclusion assay. Cells were treated as in **a**. Twenty-four and 48 hours after treatment, floating and attached cells were combined, cell suspensions were mixed with equal volume of 0.4% Trypan blue solution, and number of Trypan blue-positive cells (dead cells) was scored.

As described,^49^ human pancreatic cancer-derived AsPC-1 cells lacking *p53* were resistant to GEM. Here, we compared the effects of GEM between AsPC-1 and human pancreatic cancer SW1990 cells carrying wild-type *p53*. Toward this end, AsPC-1 and SW1990 cells were treated with the indicated concentrations of GEM. Forty-eight hours after treatment, cells were observed under phase-contrast microscope. As shown in Supplementary Figure S1, number of attached cells was apparently decreased in GEM-treated SW1990 cells but not significantly in AsPC-1 cells following GEM exposure. Under these experimental conditions, floating and attached cells were harvested and their DNA content was analyzed by fluorescence-activate cell sorting (FACS). As seen in Figure 1a, number of cells with sub-G1 DNA content was smaller in AsPC-1 cells than SW1990 cells after GEM treatment. Consistent with these observations, Trypan blue exclusion assays demonstrated that number of Trypan blue-positive cells (dead cells) is much more smaller in AsPC-1 cells than SW1990 cells in response to GEM (Figure 1b). Thus, our present results indicate that AsPC-1 cells display GEM resistance as compared with SW1990 cells.

### Expression pattern of p53 family-related gene products in response to GEM

**Figure 2:**
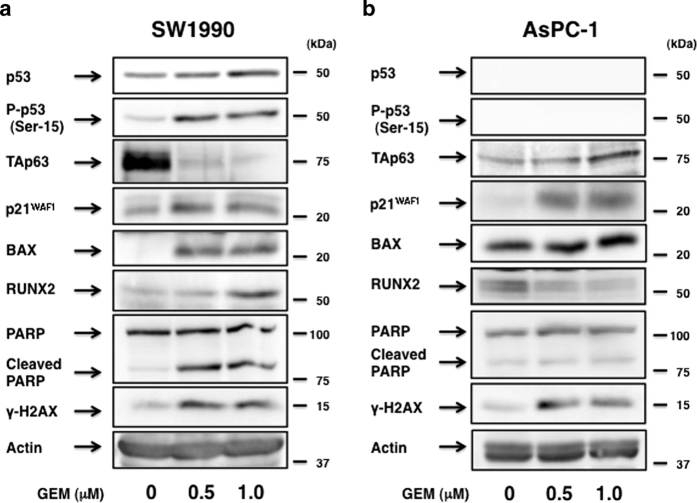
Expression analysis of p53/TAp63-related gene products in response to GEM. SW1990 (**a**) and AsPC-1 (**b**) cells were exposed to the indicated concentrations of GEM. Forty-eight hours after treatment, whole-cell lysates were prepared and subjected to immunoblotting with the indicated antibodies. Actin was used as a loading control.

To shed light on the molecular mechanism(s) behind GEM-resistant phenotype of AsPC-1 cells, we have examined the expression pattern of p53 family-related gene products following GEM exposure. In these experiments, cleavage of PARP and accumulation of γH2AX were also checked as cell death and DNA damage markers, respectively. As shown in Figure 2a, GEM-dependent robust accumulation of γH2AX and cleavage of PARP were detectable in SW1990 cells, implying that DNA damage-mediated cell death takes place. As expected, GEM exposure resulted in an induction of p53, as well as phosphorylation of p53, at Ser-15 accompanied by a stimulation of a subset of p53 family-target gene products such as p21^WAF1^ and pro-apoptotic BAX, suggesting that GEM-mediated induction of cell death of SW1990 cells is regulated at least in part in a p53-dependent manner. In addition, the expression level of p53 family member TAp63 and RUNX2 was reduced and increased in response to GEM, respectively. As RUNX2 inhibited pro-apoptotic activity of p53 following DNA damage,^47^ it is possible that GEM-mediated upregulation of RUNX2 attenuates the inappropriate cell death caused by overactive p53.

In contrast to SW1990 cells, GEM treatment led to an accumulation of γH2AX but not a promotion of PARP cleavage in AsPC-1 cells (Figure 2b). Instead of p53, GEM-dependent induction of TAp63 was observed in association with an upregulation of p21^WAF1^. Thus, it is likely that GEM-mediated induction of p21^WAF1^ may be due to TAp63. On the other hand, the expression level of BAX remained unchanged regardless of GEM treatment, whereas RUNX2 level declined in response to GEM, raising a possibility that there exists an inverse relationship between the expression level of TAp63 and RUNX2 following GEM exposure. We then focused on GEM-resistant AsPC-1 cells in our further experiments.

### TAp63 is implicated in the regulation of GEM sensitivity in AsPC-1 cells

**Figure 3:**
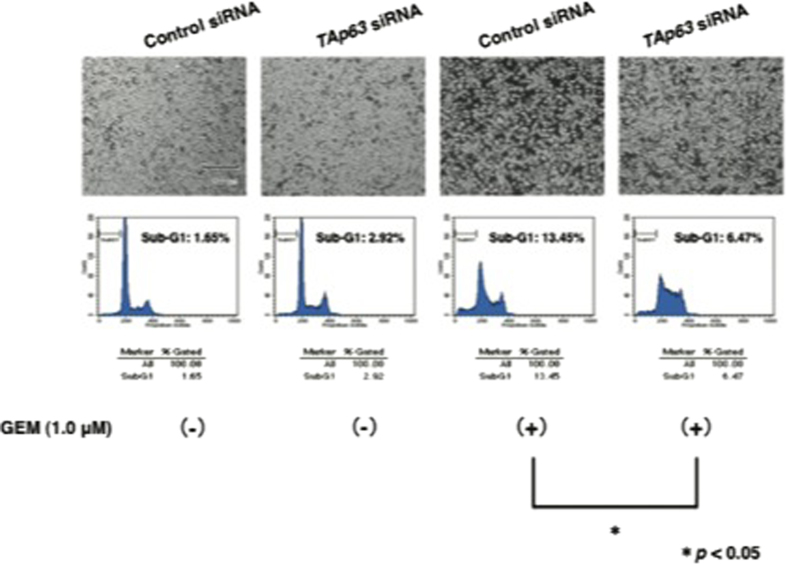
Silencing of *TAp63* lowers the sensitivity to GEM. AsPC-1 cells were transfected with control siRNA or with siRNA against *TAp63*. Twenty-four hours after transfection, cells were maintained in the presence or absence of GEM (1 *μ* M). Forty-eight hours after treatment, pictures were taken (upper panels). Under the same experimental conditions, floating and adherent cells were collected and their DNA contents were measured by FACS analysis (lower panels).

As the expression level of TAp63 was increased in AsPC-1 cells exposed to GEM, it is plausible that TAp63 may be involved in the regulation of their GEM sensitivity. To test this possibility, we sought to deplete the endogenous *TAp63*. AsPC-1 cells were transfected with control small interfering RNA (siRNA) or with siRNA against *TAp63*. These experiments were performed in the presence or in the absence of GEM. As shown in Figure 3 and Supplementary Figure S2A, FACS analysis and Trypan blue exclusion assay demonstrated that GEM sensitivity is markedly decreased in *TAp63* knockdown cells relative to non-silencing cells. These results were also supported by WST cell survival assay (Supplementary Figure S2B).

**Figure 4:**
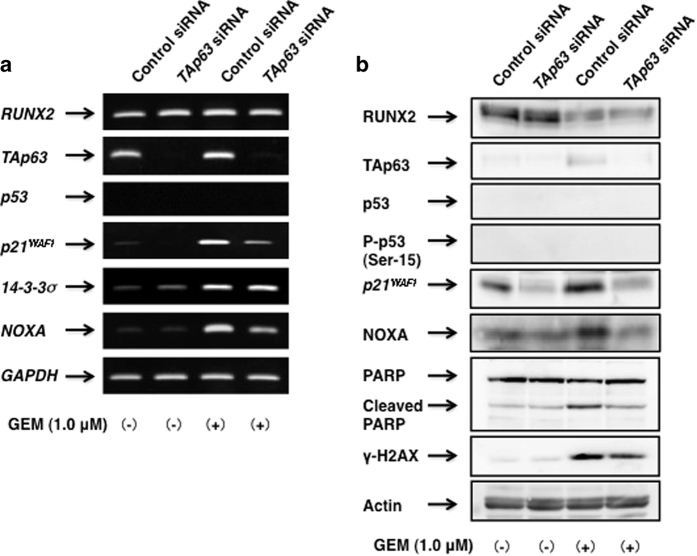
Knockdown of *TAp63* attenuates GEM-mediated induction of certain p53/TAp63-target genes. AsPC-1 cells were transfected as in Figure 3. Twenty-four hours after transfection, cells were incubated in the presence or absence of GEM (1 *μ* M) for 48 h. Total RNA and whole-cell lysates were then prepared and analyzed by RT-PCR (**a**) and immunoblotting (**b**), respectively.

These findings prompted us to examine the effects of *TAp63* silencing on GEM-dependent upregulation of p53/TAp63-target genes. For this purpose, AsPC-1 cells were transfected with control siRNA or with siRNA targeting *TAp63*. Twenty-four hours after transfection, cells were exposed to GEM or left untreated for 48 h. As shown in Figure 4a, knockdown of *TAp63* attenuated GEM-mediated induction of *p21*^*WAF1*^ and *NOXA*. Consistent with these observations, immunoblotting revealed that GEM-stimulated expression of p21^WAF1^, as well as NOXA, and cleavage of PARP are remarkably prohibited by *TAp63* depletion (Figure 4b). Together, our present results strongly suggest that TAp63-driven cell death pathway is tightly linked to GEM sensitivity of AsPC-1 cells.

### Knockdown of *RUNX2* enhances GEM sensitivity of AsPC-1 cells through the stimulation of TAp63-dependent cell death pathway

As shown in Figure 2b, there existed an inverse relationship between the expression level of TAp63 and RUNX2 in GEM-treated AsPC-1 cells, raising a possibility that RUNX2 could negatively regulate TAp63 expression. To address this issue, AsPC-1 cells were transfected with the empty plasmid or with the expression plasmid for RUNX2. As clearly seen in Supplementary Figure S3, forced expression of *RUNX2* in AsPC-1 cells resulted in a marked decrease in the expression of TAp63 at mRNA and protein levels, indicating that RUNX2 has an ability to trans-repress *TAp63*.

**Figure 5:**
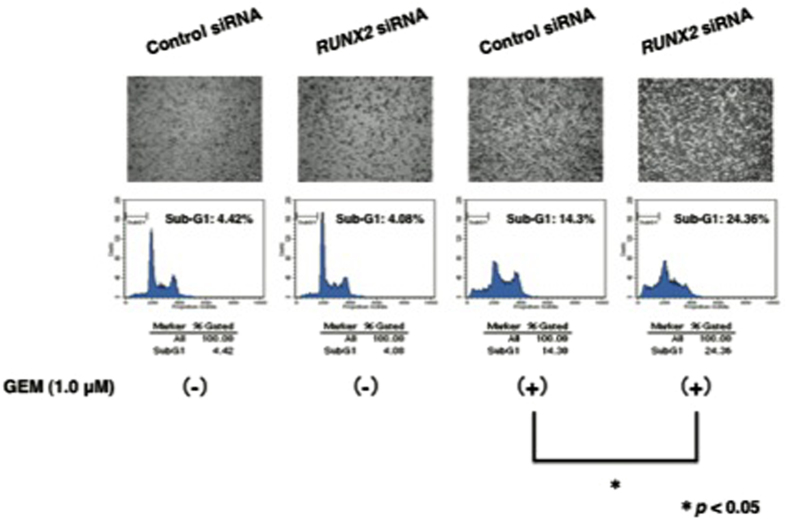
siRNA-mediated knockdown of *RUNX2* enhances GEM sensitivity of AsPC-1 cells. AsPC-1 cells were transfected with control siRNA or with siRNA against *RUNX2*. Twenty-four hours after transfection, cells were treated with GEM (1 *μ* M) or left untreated. Forty-eight hours after treatment, images were obtained with phase-contrast microscopy (upper panels). Under the same experimental conditions, floating and attached cells were collected and number of cells with sub-G1 DNA content was measured by FACS analysis (lower panels).

Considering that silencing of *RUNX2* enhances ADR sensitivity of osteosarcoma U2OS cells through the stimulation of p53/TAp73-dependent cell death pathway,^47,48^ we asked whether depletion of the endogenous *RUNX2* could augment the sensitivity to GEM in AsPC-1 cells. To this end, AsPC-1 cells were transfected with control siRNA or with siRNA targeting *RUNX2*. Transfected cells were then treated with GEM or left untreated. As shown in Figure 5 and Supplementary Figure S4A, GEM-mediated cell death was further promoted in *RUNX2* knockdown cells as compared with non-silencing control cells. These observations were supported by WST cell survival assay (Supplementary Figure S4B). Similar results were also obtained in *p53*-mutated human pancreatic cancer MiaPaCa-2 cells (Nakamura *et al.*, manuscript in preparation), indicating that *RUNX2* depletion-mediated enhancement of GEM sensitivity may not be restricted to *p53*-deficient AsPC-1 cells. Together, these results suggest that silencing of *RUNX2* augments GEM-mediated cell death in AsPC-1 cells.

**Figure 6:**
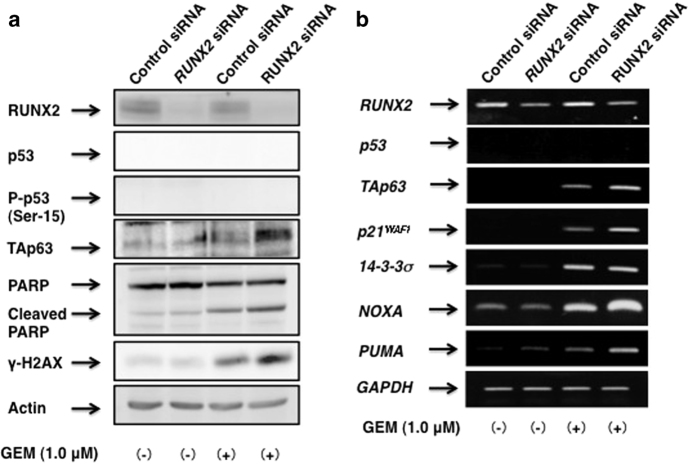
Knockdown of *RUNX2* further stimulates GEM-mediated induction of TAp63. AsPC-1 cells were transfected with control siRNA or with siRNA against *RUNX2*. Twenty-four hours after transfection, cells were treated with 1.0 *μ* M of GEM or left untreated. Forty-eight hours after treatment, whole-cell lysates and total RNA were prepared and analyzed by immunoblotting (**a**) and RT-PCR (**b**), respectively. Actin and *GAPDH* were used as a loading and an internal control, respectively.

We next sought to investigate the influence of *RUNX2* silencing on p53/TAp63-related gene expression under these experimental conditions. As expected, GEM-dependent cleavage of PARP was increased in *RUNX2* knockdown cells as compared with non-targeting siRNA-transfected cells (Figure 6a). Of note, TAp63 was strongly induced in *RUNX2* knockdown cells exposed to GEM. Not surprisingly, these observations were also supported by RT-PCR analysis. As seen in Figure 6b, depletion of *RUNX2* markedly stimulated GEM-mediated upregulation of *TAp63,* as well as a subset of p53/TAp63-target genes such as *p21*^*WAF1*^, *NOXA* and *PUMA*, indicating that silencing of *RUNX2* activates TAp63-dependent cell death pathway in response to GEM.

To further verify whether TAp63 could suppress cell proliferation and/or promote cell death in AsPC-1 cells, AsPC-1 cells were transfected with the empty plasmid or with the expression plasmid for TAp63α. As shown in Supplementary Figures S5A and B, forced expression of *TAp63α* induced a subset of p53/TAp63-target genes, whereas the expression of RUNX2 remained unchanged at mRNA and protein levels regardless of the exogenous TAp63α. In addition, AsPC-1 cells were transfected as described above and transfected cells were maintained in the presence of G418. Two weeks after selection, cells were observed under phase-contrast microscope. Three weeks after the selection, G418-resistant colonies were stained with Giemsa’s solution. As shown in Supplementary Figure S5C, number of drug-resistant colonies was markedly reduced upon transfection of TAp63α as compared with control transfection. Thus, it is conceivable that TAp63 alone is capable to suppress cell proliferation and/or induce cell death in AsPC-1 cells.

### TAp63 is involved in the regulation of GEM-dependent activation of ataxia telangiectasia-mutated protein (ATM)

**Figure 7:**
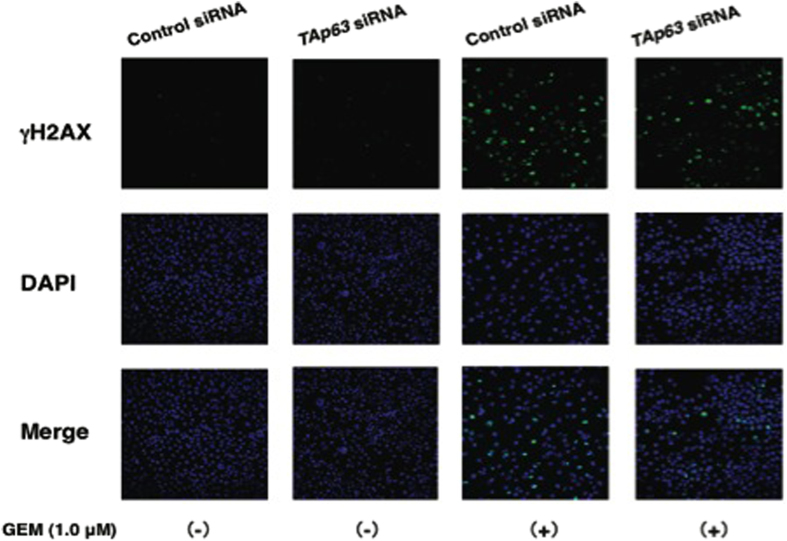
Knockdown of *TAp63* results in decreased γH2AX-positive cells in response to GEM. AsPC-1 cells were transfected as in Figure 5. Twenty-four hours after transfection, cells were treated with or without GEM (1 *μ* M) for 48 h. Cells were then incubated with anti-γH2AX antibody (green). Cell nuclei were stained with DAPI (blue).

**Figure 8:**
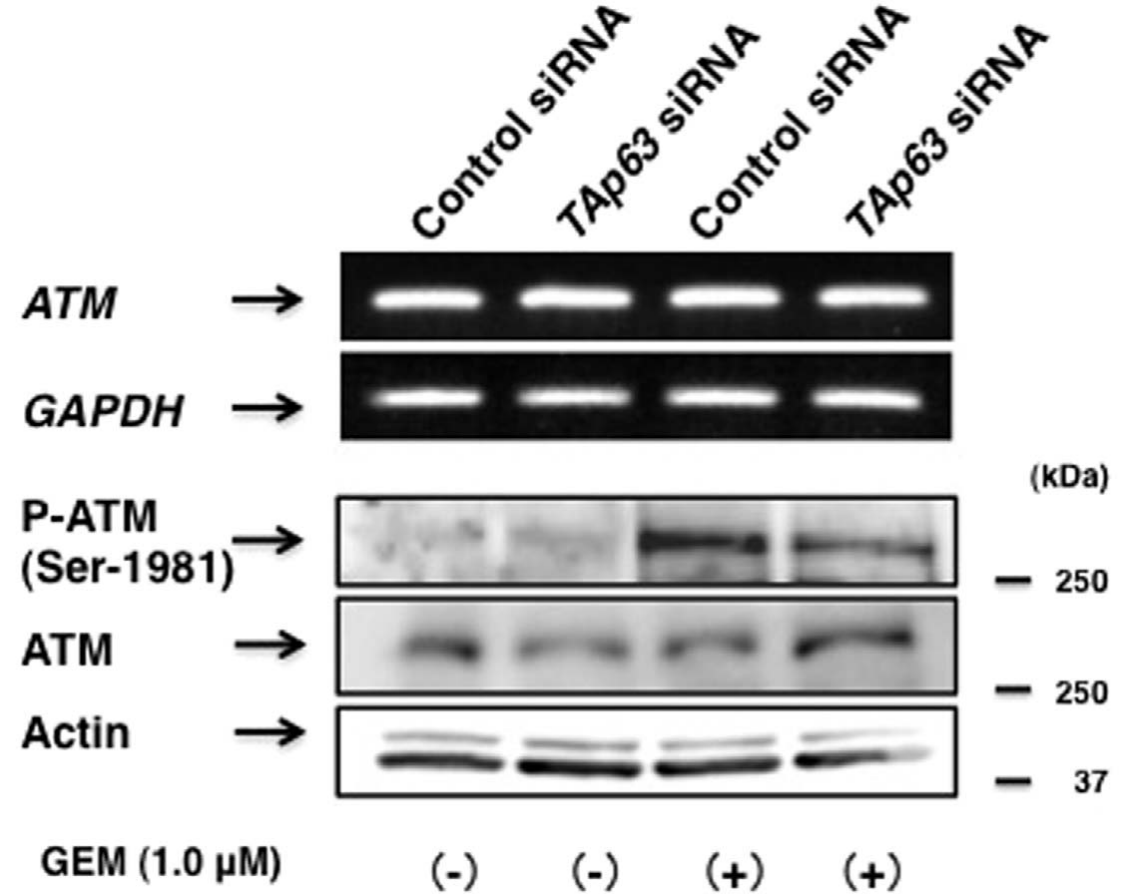
Silencing of *TAp63* prohibits GEM-dependent phosphorylation of ATM. AsPC-1 cells were transfected as in Figure 7. Twenty-four hours after transfection, cells were incubated with or without GEM (1 *μ* M) for 48 h. Total RNA and whole-cell lysates were then extracted and analyzed by RT-PCR and immunoblotting, respectively.

Close inspection of the immunoblots as shown in Figures 4b and 6a revealed that silencing of *TAp63* or *RUNX2* results in a decrease or increase in GEM-dependent accumulation of γH2AX relative to GEM-treated non-silencing cells, respectively. To further confirm these observations, AsPC-1 cells were transfected with control siRNA or with siRNA against *TAp63* followed by the incubation with or without GEM for 48 h. Cells were then fixed and stained with anti- γH2AX antibody. As clearly shown in Figure 7 and Supplementary Figure S6, knockdown of *TAp63* plus GEM treatment caused the reduction in number of γH2AX-positive cells as compared with non-silencing cells exposed to GEM. As it has been well documented that histone H2AX is phosphorylated by phospho-ATM (p-ATM) in response to DNA damage,^50^ we have assessed the effects of *TAp63* silencing on the amount of p-ATM in response to GEM. For this purpose, AsPC-1 cells were treated as in Figure 7. As expected, ATM became phosphorylated at Ser-1981 after GEM exposure (Figure 8). Intriguingly, GEM-dependent phosphorylation of ATM was markedly prohibited in *TAp63* knockdown cells. Meanwhile, total amount of ATM was basically unaffected. Collectively, these results indicate that TAp63 is required for GEM-mediated activation of ATM and/or the maintenance of the activated form of ATM.

## Discussion

As described,^51^ transcriptionally active TAp63 has an overlapping function of the other p53 family members such as TAp73 and p53. For example, TAp63 can promote cell cycle arrest and/or cell death in response to genotoxic insults. In this study, we have found for the first time that silencing of pro-oncogenic *RUNX2* in *p53*-deficient pancreatic cancer AsPC-1 cells helps to amplify TAp63 response to GEM and bolsters its tumor-suppressive function. As GEM-mediated accumulation of TAp63 in *RUNX2* knockdown cells remarkably stimulated the expression of a subset of p53/TAp63-target gene products including cell cycle-related p21^WAF1^ and pro-apoptotic NOXA, it is highly likely that RUNX2 contributes at least in part to the acquisition and/or maintenance of GEM-resistant phenotype of *p53*-deficient pancreatic cancer cells through the downregulation of TAp63-dependent cell death pathway.

It has been well documented that, unlike *p73* and *p63*, *p53* is frequently mutated in human cancer tissues.^43,44^ Especially, around 75% of pancreatic cancers carry *p53* mutations.^42^ Hamed *et al.^52^* showed that AsPC-1 cells display GEM-resistant phenotype.^51^ According to our present results, AsPC-1 cells exhibited a much more higher resistance to GEM relative to *p53*-proficient pancreatic cancer SW1990 cells, indicating that SW1990 cells but not AsPC-1 cells undergo cell death following GEM exposure in a p53-dependent manner. Of note, GEM-dependent accumulation of γH2AX was also observed in AsPC-1 cells to an extent similar to SW1990 cells as revealed by immunoblotting and immunostaining (data not shown), implying that AsPC-1 cells receive DNA damage in the presence of GEM. These observations may rule out the possibility that the transporter function, which protects cells from the accumulation of undesirable chemotherapeutic agents such as GEM,^53^ is specifically augmented in AsPC-1 cells. Therefore, it is conceivable that γH2AX-dependent downstream cell death signaling pathway(s) is suppressed in AsPC-1 cells. Although AsPC-1 cells lack functional p53, the efforts should be made to improve GEM sensitivity of *p53*-defective and/or *p53*-mutated pancreatic cancer cells.

Intriguingly, siRNA-mediated silencing of *RUNX2* positively impacted on GEM sensitivity of AsPC-1 cells, which was accompanied by a marked upregulation of TAp63 and a subset of p53/TAp63-target genes, indicating that RUNX2 suppresses TAp63-driven cell death pathway following GEM exposure. Under our experimental conditions, our siRNA against *TAp63* did not affect the expression level of pro-oncogenic ΔNp63 (data not shown). In accordance with these observations, forced expression of RUNX2 declined the expression of TAp63 at mRNA and protein levels, whereas TAp63 had an undetectable effect on RUNX2 expression. Consistent with these results, knockdown of *TAp63* lowered GEM sensitivity of AsPC-1 cells and overexpression of TAp63α gave a significant decrease in number of G418-resistant colonies as compared with control plasmid-transfected cells. These results further support the notion that TAp63 contributes to the enhancement of GEM sensitivity in AsPC-1 cells. In a good agreement with this notion, it has been shown that p53-mediated cell death is severely impaired in the absence of TAp63 or TAp73 in response to DNA damage, whereas TAp63 or TAp73 alone is able to induce cell death following DNA damage.^54^

As knockdown and overexpression of *RUNX2* increased and decreased the expression level of *TAp63*, respectively, it is likely that RUNX2 serves as a negative transcription regulator for *TAp63*. From the close inspection of 5’-upstream region of human *TAp63* gene, we could find out a putative RUNX2-binding sequence ACCACA (from –553 to –548). At present, it remains unclear whether this canonical sequence could be functional or not. It has been described that RUNX2 represses the transcription of its target genes by recruiting histone deacetylases to their promoters.^55^ Alternatively, Ng *et al.^56^* found that transcription factor OCT4 has an ability to stimulate *TAp63* but not Δ*Np63* expression at mRNA level. According to their results, the upstream region of human *TAp63* gene contained a functional OCT4-binding site (from –3044 to –3037). In addition, Park *et al.^57^* found that mono-ubiquitinated FANCD2 together with its binding partner FANCP suppresses squamous cell cancers through the activation of *TAp63* transcription. Thus, it is possible that RUNX2 may abrogate OCT4- and/or FANCD2/FANCP-mediated transcriptional activation of *TAp63*. Further studies should be required to adequately address this issue.

Another finding coming out of this study was that RUNX2/TAp63 regulatory axis is strongly involved in the regulation of GEM-mediated activation of ATM. According to our present results, knockdown of *RUNX2* or *TAp63* resulted in GEM-dependent increase or decrease in the amounts of γH2AX, respectively. Moreover, GEM-mediated phosphorylation (activation) of ATM was robustly reduced in *TAp63*-depleted cells as compared with non-silencing cells. Consistently, GEM-mediated cell death was prohibited in *TAp63* knockdown cells relative to non-silencing cells. Wilhelm *et al.^58^* described that ΔNp73 binds to DNA damage sensor protein 53BP1 and thereby inhibits ATM activation without direct interaction with ATM. Based on their results, cisplatin-mediated phosphorylation of H2AX was enhanced in Δ*Np73*-deficient mouse embryonic fibroblasts as compared with wild-type ones. As ΔNp73 displays a dominant-negative behavior against TAp73,^41^ it is plausible that TAp73 may have an ability to stimulate ATM activity in response to DNA damage. However, the precise molecular mechanism(s) how TAp73 could enhance ATM activity remains elusive. Under our experimental conditions, p-ATM was induced following GEM exposure in association with upregulation of TAp63, whereas ΔNp63 remained unchanged regardless of GEM treatment (data not shown). These observations indicate that the intracellular balance between TAp63 and ΔNp63 may affect GEM-dependent activation of ATM. Further studies should be required to elucidate the exact role of TAp63 in the modulation of ATM phosphorylation in response to DNA damage.

Taken together, our present findings strongly suggest that silencing of pro-oncogenic *RUNX2* enhances GEM sensitivity of *p53*-deficient AsPC-1 cells through the upregulation of pro-apoptotic TAp63 and may be an attractive therapeutic strategy for the treatment of the patients with GEM-resistant malignant pancreatic cancer.

## Materials and Methods

### Cells and transfection

Human pancreatic cancer-derived SW1990 and AsPC-1 cells (ATCC, Manassas, VA, USA) were maintained in Dulbecco’s modified Eagle’s medium (Wako, Osaka, Japan) supplemented with heat-inactivated 10% fetal bovine serum (Invitrogen, Carlsbad, CA, USA) and penicillin–streptomycin at 37 °C in 5% CO_2_. For transfection, cells were transfected with the indicated expression plasmids using Lipofectamine 2000 transfection reagent according to the manufacturer’s instructions (Invitrogen).

### Immunoblotting

GEM-treated cells were harvested in lysis buffer (25 mM Tris-HCl, pH 8.0, 137 mM NaCl, 2.7 mM KCl and 1% Triton X-100) supplemented with a commercial protease inhibitor mixture (Sigma, St. Louis, MO, USA). Whole-cell lysates were analyzed by immunoblotting as described,^47^ using the following antibodies: anti-p53 (DO-1, Santa Cruz Biotechnology, Santa Cruz, CA, USA), anti-phospho-p53 at Ser-15 (Cell Signaling, Beverly, MA, USA), anti-p63 (4A4, NeoMarkers, Fremont, CA, USA), anti-p21^WAF1^ (H-164, Santa Cruz Biotechnology), anti-BAX (Cell Signaling), anti-NOXA (114C307, Abcam, Cambridge, MA, USA), anti-RUNX2 (Cell Signaling), anti-PARP (Cell Signaling), anti-γH2AX (2F3, BioLegend, San Diego, CA, USA), anti-ATM (5C2, Santa Cruz Biotechnology), anti-phospho-ATM at Ser-1981 (Cell Signaling) and anti-actin (20-33, Sigma).

### WST assay

Cell were seeded at a density of 1×10^3^ cells/96-well plates and allowed to attach overnight. Cells were then treated with the indicated concentrations of GEM. Forty-eight hours after treatment, 10 *μ*l of a modified 3-(4,5-dimethylthiazol-2-yl) 2,5-diphenyl-tetrazolium bromide solution (Dojindo, Kumamoto, Japan) were added to the culture and reaction mixtures were incubated at 37°C for 2 h. The absorbance readings for each well were carried out at 570 nm using the microplate reader (Model 450, Bio-Rad, Hercules, CA, USA).

### FACS analysis

For flow cytometric analysis, cells were fixed in 70% ethanol at –20°C overnight, then incubated with 1 mg/ml of RNase A at 37°C for 30 min and stained with 50*μ*g/ml of propidium iodide. The DNA contents were analyzed by a FACScan flow cytometer (Becton Dickinson, Lincoln Park, NJ, USA).

### Trypan blue exclusion assay

Floating and trypsinized attached cells were collected and suspended in fresh medium at room temperature. Twenty microliters of cell suspension were mixed with 20 *μ*l of 0.4% Trypan blue solution (Bio-Rad), and 10 *μ*l of this mixture were used for counting blue (dead) and white (alive) cells using TC20 automated cell counter (Bio-Rad).

### RT-PCR

Total RNA extracted from the indicated cells using RNeasy mini kit (Qiagen, Valencia, CA, USA) was subjected to semiquantitative RT-PCR using SuperScript II reverse transcriptase and random primers (Invitrogen) according to the manufacturer’s instructions. Oligonucleotide primer sets used in this study were as follows: *p53*, 5′-CTGCCCTCAACAAGATGTTTTG-3′ (forward) and 5′-CTATCTGAGCAGCGCTCATGG-3′ (reverse); *TAp63*, 5′-GACCTGAGTGACCCCATGTG-3′ (forward) and 5′-CGGGTGATGGAGAGAGAGCA-3′ (reverse); *RUNX2*, 5′-TCTGGCCTTCCACTCTCAGT-3′ (forward) and 5′-GACTGGCGGGGTGTAAGTAA-3′ (reverse); *p21*^*WAF1*^, 5′-ATGAAATTCACCCCCTTTCC-3′ (forward) and 5′-CCCTAGGCTGTGCTCACTTC-3′ (reverse); *14-3-3σ*, 5′-GAGCGAAACCTGCTCTCAGT-3′ (forward) and 5′-CTCCTTGATGAGGTGGCTGT-3′ (reverse); *NOXA*, 5′-CTGGAAGTCGAGTGTGCTACT-3′ (forward) and 5′-TCAGGTTCCTGAGCAGAAGAG-3′ (reverse); *PUMA*, 5′-GCCCAGACTGTGAATCCTGT-3′ (forward) and 5′-TCCTCCCTCTTCCGAGATTT-3′ (reverse); *BAX*, 5′-AGAGGATGATTGCCGCCGT-3′ (forward) and 5′-CAACCACCCTGGTCTTGGAT-3′ (reverse); glyceraldehyde 3-phosphate dehydrogenase (*GAPDH*), 5′-ACCTGACCTGCCGTCTAGAA-3′ (forward) and 5′-TCCACCACCCTGTTGCTGTA-3′ (reverse).

### Colony formation assay

AsPC-1 cells were transfected with the empty plasmid (pcDNA3) or with the expression plasmid for TAp63α. Forty-eight hours after transfection, cells were transferred into fresh medium containing G418 (400 *μ*g/ml). Two weeks after the selection, cells were observed under phase-contrast microscope. Three weeks after the selection, G418-resistant colonies were fixed and stained with Giemsa’s solution.

### Immunostaining

Immunostaining was performed by fixing cells in 3.7% formaldehyde at room temperature for 30 min followed by permeabilization with 0.1% Triton X-100 in PBS for 5 min. Cells were then blocked with 3% BSA in PBS at room temperature for 1 h. After blocking, cells were incubated with anti-γH2AX antibody at room temperature for 1 h. Cells were washed in PBS and incubated with fluorescein isothiocyanide-conjugated goat anti-mouse IgG (Invitrogen) at room temperature for 1 h. After incubation, cells were extensively washed in PBS and the coverslips were mounted on glass slides with VectaShield (Vector Laboratories, Peterborough, UK). Images were captured using a confocal laser scanning microscope (Leica, Wetzlar, Germany). Cell nuclei were stained with 4′,6-diamidino-2-phenylindole (DAPI).

### Statistical analysis

All cell-based assays described in this study were performed independently at least three times. The data in all graphs were analyzed with Microsoft Excel (Microsoft Co., Redmond, WA, USA) and represented means±S.D. from two independent experiments performed in triplicates. Statistical significance was determined with Student’s *t*-test, and *P*-values <0.05 were considered statistically significant.

## Abbreviations

ADR: adriamycin
ATM: ataxia telangiectasia-mutated protein
DAPI: 4′,6-diamidino-2-phenylindole
FACS: fluorescence-activate cell sorting
GAPDH: glyceraldehyde 3-phosphate dehydrogenase
GEM: gemcitabine
PARP: poly ADP ribose polymerase
RUNX2: runt-related transcription factor 2
siRNA: small interfering RNA.
