# Supplementary material for: Silencing of RUNX2 enhances gemcitabine sensitivity of p53-deficient human pancreatic cancer AsPC-1 cells through the stimulation of TAp63-mediated cell death
Source: Cell Death Discov. 2015 Aug 10;1:15010–. doi: 10.1038/cddiscovery.2015.10 (PMC4981025; doi:10.1038/cddiscovery.2015.10)
Supplement: Supplementary Figures [file cddiscovery201510-s1.doc]

**Figure S1** Effect of GEM on SW1990 and AsPC-1 cell proliferation. SW1990 (upper panels) and AsPC-1 (lower panels) cells treated as in Figure 1a. Forty-eight hours after treatment, cells were observed under phase-contrast microscopy and representative pictures were taken. Scale bar, 300 μm.

**Figure S2** Depletion of *RUNX2* reduces and stimulates cell viability and cell death following GEM exposure, respectively. AsPC-1 cells were transfected with control siRNA or with siRNA targeting *RUNX2*. Twenty-four hours after transfection, cells were maintained in the presence or absence of GEM (1 μM) for 48 h. Cells were then processed for MTT cell survival assay (**a**). Under the same experimental conditions, floating and attached cells were collected and subjected to trypan blue exclusion assay (**b**).

**Figure S3** Forced expression of RUNX2 reduces TAp63. AsPC-1 cells were transfected with the empty plasmid (pcDNA3) or with the expression plasmid encoding RUNX2. Forty-eight hours after transfection, total RNA and whole cell lysates were prepared and analyzed by RT-PCR (**a**) and immunoblotting (**b**), respectively.

**Figure S4** Forced expression of TAp63 transactivates certain p53-target genes and inhibits AsPC-1 cell proliferation. AsPC-1 cells were transfected with the empty plasmid (pcDNA3) or with the expression plasmid for TAp63α. Forty-eight hours after transfection, total RNA and whole cell lysates were prepared and analyzed by RT-PCR (**a**) and immunoblotting (**b**), respectively. Alternatively, transfected cells were transferred into fresh medium containing 400 μg/ml of G418. Two weeks after the selection, pictures were taken (**c**).

**Figure S5** Knockdown of *TAp63* results in an increase and a decrease in cell viability and number of dead cells in response to GEM, respectively. AsPC-1 cells were transfected with control siRNA or with siRNA against *TAp63*. Twenty-four hours after transfection, cells were exposed to 1 μM of GEM or left untreated. Forty-eight hours after treatment, cell viability was assessed by MTT assay (**a**). Under the same experimental conditions, floating and attached cells were harvested and subjected to trypan blue exclusion assay (**b**).

**Figure S6** Depletion of *TAp63* reduces number of γH2AX-positive cells in response to GEM. AsPC-1 cells were transfected as in Figure S5. Twenty-four hours after transfection, cells were exposed to GEM (1 μM) or left untreated for 48 h. cells were then incubated with anti-γH2AX and number of γH2AX-positive cells was scored.
