# Supplementary figures and images for: Silencing of RUNX2 enhances gemcitabine sensitivity of p53-deficient human pancreatic cancer AsPC-1 cells through the stimulation of TAp63-mediated cell death
Source: Cell Death Discov. 2015 Aug 10;1:15010–. doi: 10.1038/cddiscovery.2015.10 (PMC4981025; doi:10.1038/cddiscovery.2015.10)

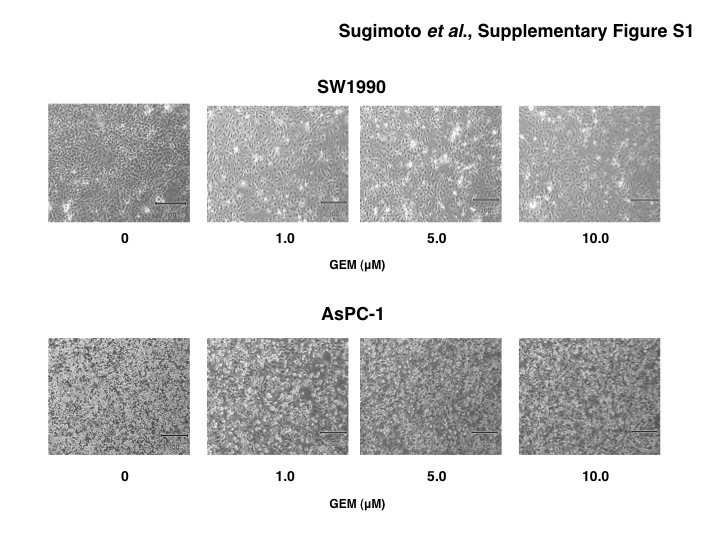

Supplement: Supplementary Figure 1 [file cddiscovery201510-s2.jpg]

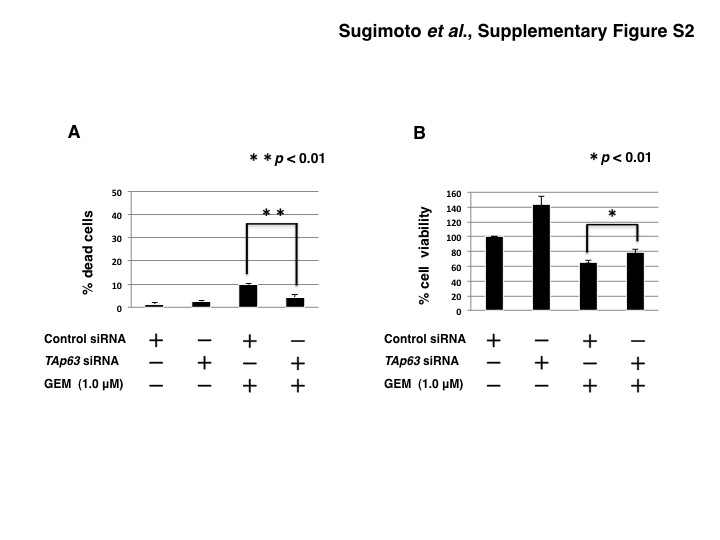

Supplement: Supplementary Figure 2 [file cddiscovery201510-s3.jpg]

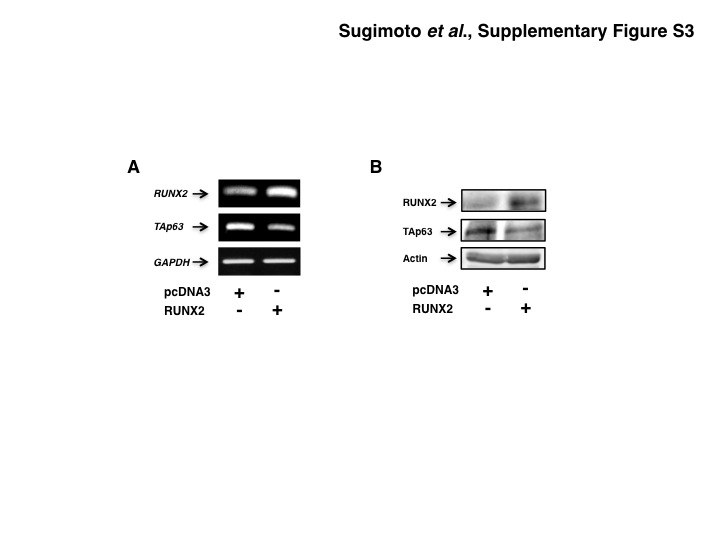

Supplement: Supplementary Figure 3 [file cddiscovery201510-s4.jpg]

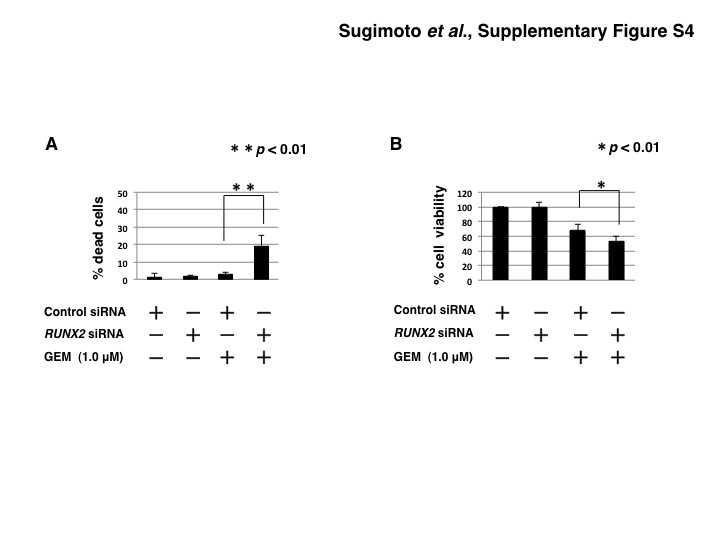

Supplement: Supplementary Figure 4 [file cddiscovery201510-s5.jpg]

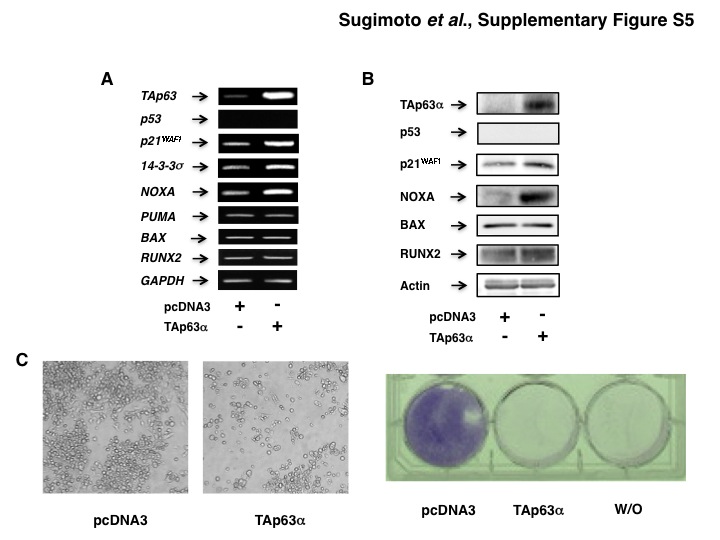

Supplement: Supplementary Figure 5 [file cddiscovery201510-s6.jpg]

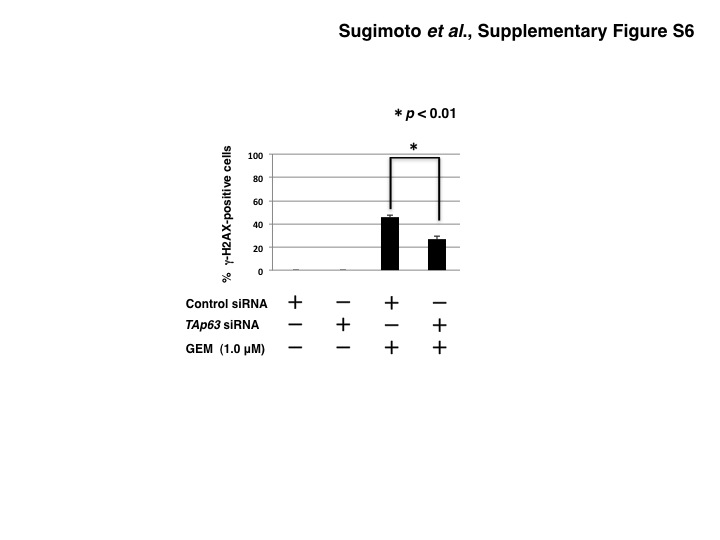

Supplement: Supplementary Figure 6 [file cddiscovery201510-s7.jpg]
